# Clinical Evidence for Association of Acupuncture and Acupressure With Improved Cancer Pain: A Systematic Review and Meta-Analysis

**DOI:** 10.1001/jamaoncol.2019.5233

**Published:** 2019-12-19

**Authors:** Yihan He, Xinfeng Guo, Brian H. May, Anthony Lin Zhang, Yihong Liu, Chuanjian Lu, Jun J. Mao, Charlie Changli Xue, Haibo Zhang

**Affiliations:** 1 Guangdong Provincial Hospital of Chinese Medicine, The Second Affiliated Hospital of Guangzhou University of Chinese Medicine, Guangdong Provincial Academy of Chinese Medical Sciences, Guangzhou, Guangdong Province, China; 2 China-Australia International Research Centre for Chinese Medicine, School of Health and Biomedical Sciences, RMIT University, Melbourne, Victoria, Australia; 3 Integrative Medicine Department, Memorial Sloan Kettering Cancer Center, New York, New York

## Abstract

**Importance:**

Research into acupuncture and acupressure and their application for cancer pain has been growing, but the findings have been inconsistent.

**Objective:**

To evaluate the existing randomized clinical trials (RCTs) for evidence of the association of acupuncture and acupressure with reduction in cancer pain.

**Data Sources:**

Three English-language databases (PubMed, Embase, and CINAHL) and 4 Chinese-language biomedical databases (Chinese Biomedical Literature Database, VIP Database for Chinese Technical Periodicals, China National Knowledge Infrastructure, and Wanfang) were searched for RCTs published from database inception through March 31, 2019.

**Study Selection:**

Randomized clinical trials that compared acupuncture and acupressure with a sham control, analgesic therapy, or usual care for managing cancer pain were included.

**Data Extraction and Synthesis:**

Data were screened and extracted independently using predesigned forms. The quality of RCTs was appraised with the Cochrane Collaboration risk of bias tool. Random-effects modeling was used to calculate the effect sizes of included RCTs. The quality of evidence was evaluated with the Grading of Recommendations Assessment, Development and Evaluation approach.

**Main Outcomes and Measures:**

The primary outcome was pain intensity measured by the Brief Pain Inventory, Numerical Rating Scale, Visual Analog Scale, or Verbal Rating Scale.

**Results:**

A total of 17 RCTs (with 1111 patients) were included in the systematic review, and data from 14 RCTs (with 920 patients) were used in the meta-analysis. Seven sham-controlled RCTs (35%) were notable for their high quality, being judged to have a low risk of bias for all of their domains, and showed that real (compared with sham) acupuncture was associated with reduced pain intensity (mean difference [MD], −1.38 points; 95% CI, −2.13 to −0.64 points; *I*^2^ = 81%). A favorable association was also seen when acupuncture and acupressure were combined with analgesic therapy in 6 RCTs for reducing pain intensity (MD, −1.44 points; 95% CI, −1.98 to −0.89; *I*^2^ = 92%) and in 2 RCTs for reducing opioid dose (MD, −30.00 mg morphine equivalent daily dose; 95% CI, −37.5 mg to −22.5 mg). The evidence grade was moderate because of the substantial heterogeneity among studies.

**Conclusions and Relevance:**

This systematic review and meta-analysis found that acupuncture and/or acupressure was significantly associated with reduced cancer pain and decreased use of analgesics, although the evidence level was moderate. This finding suggests that more rigorous trials are needed to identify the association of acupuncture and acupressure with specific types of cancer pain and to integrate such evidence into clinical care to reduce opioid use.

## Key Points

**Question:**

Is the use of acupuncture and acupressure associated with improved cancer pain management compared with sham intervention and/or analgesic therapy alone?

**Findings:**

In this systematic review of 17 randomized clinical trials and meta-analysis of 14 trials in the current English-language and Chinese-language literature, a significant association was found between real (compared with sham) acupuncture and reduced pain, and acupuncture combined with analgesic therapy was associated with decreased analgesic use. However, heterogeneity lowered the level of certainty of the evidence.

**Meaning:**

This study found a moderate level of evidence that acupuncture and/or acupressure was significantly associated with lower pain intensity in patients with cancer compared with a sham control, which suggests a potential for a combination of acupuncture and acupressure to help reduce opioid doses in patients with cancer.

## Teaser

This systematic review and meta-analysis provides an updated synthesis of the published randomized clinical trials into the use and advantages of acupuncture and/or acupressure among patients with cancer pain.

## Introduction

Pain is a distressing symptom experienced by more than 70% of patients with cancer but inadequately controlled in nearly 50% of these patients.^1,2^ Although the World Health Organization analgesic ladder provides effectual approaches to relieve cancer pain,^3^ addiction to analgesics and the adverse effects of pharmacological interventions pose critical challenges to pain management.^4,5^ Because pain is multidimensional, a multidisciplinary, comprehensive approach is needed to control pain successfully.^6,7^

The ongoing opioid crisis in the United States exacerbates the challenges surrounding cancer pain management,^8,9,10,11^ with government organizations calling for the use of nonpharmacological interventions^12^ on the basis of increasing clinical evidence.^13,14^ Leading organizations in the field, such as the American Society for Clinical Oncology and the National Comprehensive Cancer Network, recommend nonpharmacological interventions, such as acupuncture, for managing cancer pain.^15,16^

Research on acupuncture for cancer pain has been growing, but the findings have been inconsistent.^17^ Although more than 20 systematic reviews were conducted to establish the association of acupuncture with cancer pain,^18,19,20,21,22,23,24,25,26,27,28,29,30,31,32,33,34,35,36,37,38,39,40^ none arrived at a definitive conclusion. In addition, more rigorous randomized clinical trials (RCTs) of acupuncture and related therapies published in recent years were not included in previous systematic reviews. For example, a multicenter RCT of acupuncture published in 2018 found that patients with early-stage breast cancer who received aromatase inhibitor therapy experienced significant pain relief.^41^ The reduction in pain reported in this 2018 study was clinically relevant.^42^

In light of the growing number of RCTs of acupuncture or acupressure use for cancer pain and the ensuing need for critical evaluation, we conducted a systematic review and meta-analysis of the available evidence to inform clinical practice. The specific research questions were as follows: (1) Are acupuncture and acupressure associated with reduction in cancer pain compared with sham control or usual care control? (2) Is the combination of acupuncture and acupressure associated with reduction in analgesic use in patients with cancer?

## Methods

This systematic review and meta-analysis (PROSPERO registration No. CRD42017064113), with its peer-reviewed protocol published online,^43^ focused on RCTs involving acupuncture and acupressure interventions for addressing pain intensity and analgesic dose in patients with cancer. The quality of RCTs was appraised with the Cochrane Collaboration risk of bias tool.^44^ The overall evidence and certainty of evidence were evaluated with the Grading of Recommendations Assessment, Development and Evaluation approach.

### Search Strategy and Study Selection

Three English-language databases (PubMed, Embase, and CINAHL) and 4 Chinese-language databases (Chinese Biomedical Literature Database, VIP Database for Chinese Technical Periodicals, China National Knowledge Infrastructure, and Wanfang) were searched for RCTs published from the database inception through March 31, 2019. The search strategy consisted of 3 components: clinical condition (cancer, tumor/tumour, carcinoma, neoplasm AND pain, analgesia), intervention (manual acupuncture, electroacupuncture, body or auricular acupressure), and study type (randomized clinical trial). Existing systematic reviews were examined to identify additional trials. Two of us (Y.H. and Y.L.) independently screened the records of comprehensive searches by titles and abstracts, or full text as needed, to establish the eligibility of the studies. Articles published in the English or Chinese language were included if they were RCTs (with or without blinding, including crossover design and pragmatic trials) investigating the association of acupuncture and acupressure with cancer pain. Pain directly accompanying the development of cancer and/or chronic pain associated with cancer treatments was included. Studies of acupuncture for short-term analgesia associated with surgical procedures were excluded. Eligible interventions were acupuncture and acupressure regardless of needling techniques and stimulation methods, including manual acupuncture and acupressure, electroacupuncture, or a combination of these techniques. The comparison could be between sham or placebo acupuncture and analgesic therapy or usual care without additional intervention. Studies comparing 2 kinds of acupuncture techniques or acupuncture with another traditional Chinese medicine therapy (eg, herbal medicine, massage) were excluded. Pain intensity was selected as the targeted outcome because of its substantial role in cancer pain assessment and pain management.^45^ Outcome measures included the Brief Pain Inventory, Numerical Rating Scale, Visual Analog Scale, Verbal Rating Scale, and other validated instruments for assessing the intensity of pain. Studies that reported only improvement rates were excluded.

### Data Extraction and Quality Assessment

All data extraction was independently undertaken by 2 of us (Y.H. and X.G.) using predesigned forms. Clinical features (participants, interventions, and outcome measurements), details of the treatments, methodological characteristics, and the results of each outcome were extracted for each study. Two of us (Y.H. and Y.L.) independently appraised the quality of studies included, and disagreements were resolved by discussion and consensus with another reviewer (A.L.Z.). Each RCT was assigned a low, high, or unclear risk of bias for 6 specific domains (sequence generation, allocation concealment, blinding of participants and outcome assessment, incomplete outcome data, selective outcome reporting, and other potential threats), using information identified from the published articles and supplementary materials and by contacting the study authors when needed.

### Synthesis of Evidence

Meta-analysis of RCTs with available data was performed by calculating the effect size and 95% CI using the random-effects model. Heterogeneity among trials was identified by the χ^2^ test and reported as *I*^2^. Statistical analyses were performed with Stata, version 14.0 (StataCorp LLC). Two-sided *P* < .05 was considered statistically significant.

Studies were grouped according to the type of intervention (acupuncture, acupressure, or combination) and the comparator (sham therapy). For studies with more than 1 control group, such as real acupuncture vs sham acupuncture vs wait- list control, the results were split into pairwise comparisons by the different comparators. When different outcomes from the same study were reported in separate publications, the data were merged.

Given the strong correlation between scales of pain assessment,^46^ the results measured by the Visual Analog Scale were converted to the corresponding grade in the 11-point Numerical Rating Scale (0 points indicating no pain and 10 points indicating most severe pain). For example, a result of 40 mm on the 100-mm version of the Visual Analog Scale was recorded as 4 points for data synthesis. The results of the Numerical Rating Scale, the converted Visual Analog Scale, and the Brief Pain Inventory severity subscale were used in the meta-analysis.

Subgroup sensitivity analyses were conducted to explore potential sources of heterogeneity. When possible and appropriate, planned subgroup analyses included the source of pain (cancerous organ or specific treatment), severity of pain (mild, moderate, or severe^16^), and type of treatment (manual acupuncture, electroacupuncture, or auricular acupuncture).

Publication bias was assessed by funnel plots and the Egger test for asymmetry when at least 10 trials were included. The quality and certainty of evidence are summarized in the eTable.

## Results

**Figure 1.**
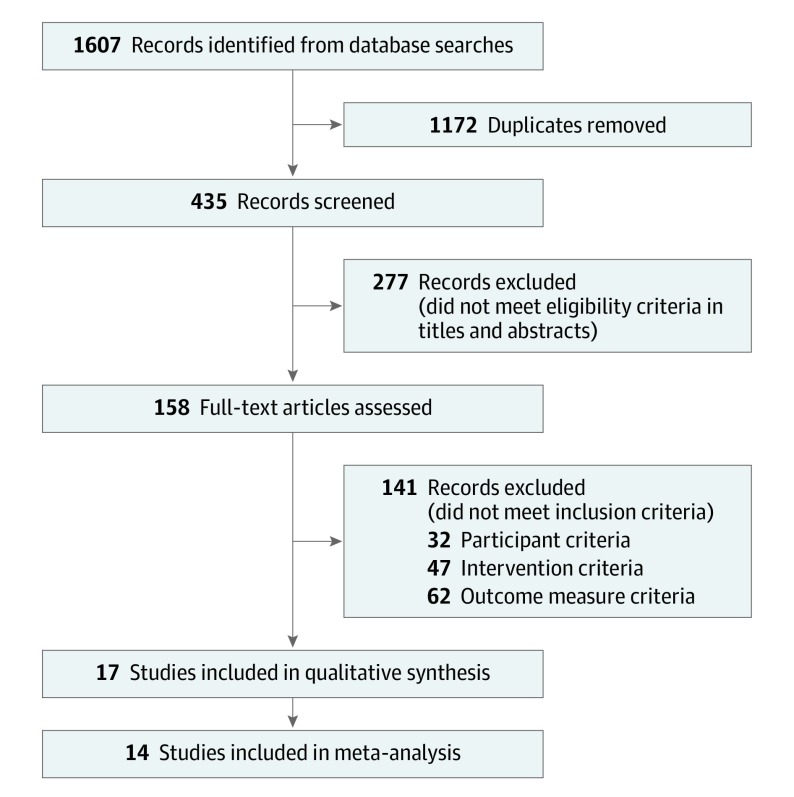
Flow Diagram

A total of 1607 articles were identified through database searches, from which 1172 duplicate publications (73%) were removed and 418 articles (26%) were excluded for not meeting the inclusion criteria. Seventeen RCTs (1%) were included in the systematic review or qualitative synthesis^41,47,48,49,50,51,52,53,54,55,56,57,58,59,60,61,62^ (Figure 1 and Table). The study characteristics of these RCTs are summarized in the eTable in the Supplement. Quantitative synthesis was performed with 14 RCTs (82%) by pooling the results through a meta-analysis; these 14 trials involved 920 patients with cancer. Three (18%) of the 17 trials had insufficient data.^55,57,61^ Seven of the studies (41%) included were conducted in China, 6 (35%) were in the United States, and 1 (6%) each were in Australia, Brazil, France, and Korea.

**Table.** Summary of Findings

Certainty Assessment							No. of Patients		Effect Size	Level of Certainty
No. of Studies	Source	Risk of Bias	Inconsistency	Indirectness	Imprecision	Publication Bias	Acupuncture and/or Acupressure Group	Control Group	Mean Difference (95% CI)	
Real vs sham acupuncture for reducing pain intensity										
7	Hershman et al,^41^ 2018; Ruela et al,^47^ 2018; Kim and Lee,^48^ 2018; Mao et al,^53^ 2014; Chen et al,^56^ 2013; Crew et al,^60^ 2010; Alimi et al,^62^ 2003	Not serious	Serious^a^	Not serious	Not serious	Undetected	226	172	−1.38 points (−2.13 to −0.64)	Moderate
Acupuncture and/or acupressure plus analgesics vs analgesics only for reducing pain intensity										
6	Wang et al,^49^ 2017; Shen et al,^50^ 2016; Wang et al,^51^ 2015; Guo et al,^52^ 2015; Zhu et al,^54^ 2013; Jiang,^58^ 2011	Serious^b^	Serious^c^	Not serious	Not serious	Undetected	195	195	−1.44 points (−1.98 to −0.89)	Low
Acupuncture vs wait-list control for reducing pain intensity										
3	Hershman et al,^41^ 2018; Mao et al,^53^ 2014; Pfister et al,^59^ 2010	Serious^b^	Not serious	Not serious	Not serious	Undetected	151	104	−1.63 points (−2.14 to −1.13)	Moderate
Acupuncture and/or acupressure plus analgesics vs analgesics only for reducing analgesic dose										
2	Wang et al,^49^ 2017; Zhu et al,^54^ 2013	Serious^b^	Not serious	Not serious	Not serious	Undetected	53	53	−30.00 mg of morphine equivalent daily dose (−37.5 to −22.5)	Moderate

^a^ Heterogeneity: *I*^2^ = 81%.

^b^ High risk of performance and detection bias owing to nonblinding.

^c^ Heterogeneity: *I*^2^ = 92%.

### Characteristics of Clinical Studies and Quality of Evidence

Among the 17 RCTs included, 9 (53%) were sham controlled and 8 (47%) were open-label trials. Thirteen studies (76%) employed a 2-group parallel design, 6 of which compared real acupuncture with sham acupuncture^47,48,55,56,57,60^ and 7 of which compared the combination of acupuncture and acupressure with analgesic therapy^49,50,51,52,54,58^ or usual care.^59^ Three 3-group studies (18%) were included; 2 compared real acupuncture with sham acupuncture or wait-list control^41,53^ and 1 compared acupuncture with 2 kinds of sham control.^62^ One RCT (6%) assessed the outcome of acupuncture using an open-label crossover design.^61^ Sample sizes ranged from 21 to 226 patients, and a total of 1111 patients were included, with 515 (46%) in the experimental group, 575 (52%) in the control group, and 21 (2%) in the crossover RCT.

Thirteen studies (76%) focused on a specific kind of cancer pain (6 aromatase inhibitor–induced arthralgia,^41,53,55,57,60,61^ 2 lung cancer pain,^49,50^ 1 gastric cancer pain,^52^ 1 pancreatic cancer pain,^56^ 1 malignant neuropathic pain,^54^ 1 osseous metastatic pain,^51^ and 1 persistent pain after a surgical procedure^59^), and 4 (24%) studies investigated general cancer pain with a mix of cancer diagnoses.^47,48,58,62^ The inclusion criteria in 13 studies limited pain to moderate and severe (at least 3 or 4 points on a 0-to-10 scale) intensity.^41,47,50,51,53,54,55,56,57,59,60,61,62^ A detailed summary of participants is provided in the eTable in the Supplement. Six sham-controlled studies (35%) were notable for their high quality, as each of the 6 domains in these studies was judged to have a low risk of bias.^41,48,53,55,57,60^ Because the measurements for pain were subjective patient-reported outcomes, detection bias existed if participants were not blinded to the treatments. Therefore, the 7 open-label, 2-group RCTs (41%) without sham acupuncture were rated as having a high risk of bias for blinding of the participants and outcome assessors.^49,50,51,52,54,58,59^ For the 3-group studies that compared real acupuncture with sham acupuncture or wait-list controls, blinding was rated as having a low risk of bias for the former comparison and a high risk of bias for the latter.^41,53^ Two studies (12%)^49,61^ were unclear about random sequence generation, and 9 (53%)^47,49,50,51,52,54,56,58,61^ were unclear on allocation concealment. Fifteen studies (88%) were at low risk of attrition bias, and 10 (59%) were at low risk of selective outcome reporting (eTable in the Supplement).

### Outcomes of Acupuncture and Acupressure

**Figure 2.**
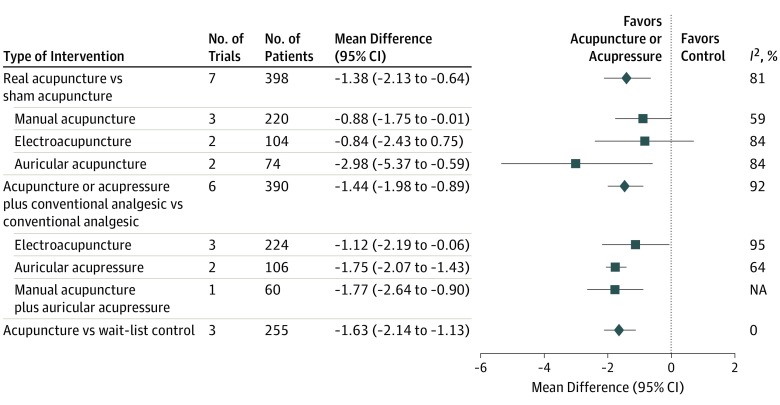
Forest Plot of the Estimated Association of Acupuncture and Acupressure With Cancer Pain Intensity The squares show the results of each subgroup analysis, and the diamond indicates the pooled effect size of all subgroups. NA indicates not applicable.

With regard to pain intensity (Figure 2), pooled results from 7 blinded studies showed the association between pain reduction and real acupuncture rather than between pain reduction and sham acupuncture with substantial heterogeneity (mean difference, −1.38 points; 95% CI, −2.13 to −0.64; *I*^2^ = 81%). Data from the 6 open-label RCTs showed the reduction in pain intensity was associated with a combination of acupuncture and acupressure when compared with analgesic therapy with considerable heterogeneity (mean difference, −1.44 points; 95% CI, −1.98 to −0.89; *I*^2^ = 92%). Significant reduction without heterogeneity was found in 3 studies that compared acupuncture with wait-list controls (mean difference, −1.63 points; 95% CI, −2.14 to −1.13) (Figure 2). Two open-label studies^49,54^ reported the maintenance dose of analgesics during the trial, and the pooled results showed a significant decrease in analgesic dose in the integrative medicine group (acupuncture plus analgesic therapy) compared with the control group that received analgesics alone (mean difference, −30.00 mg morphine equivalent daily dose; 95% CI, −37.5 mg to −22.5 mg), without heterogeneity.

In the subgroup analyses for intervention type (Figure 2), the pooled result of sham-controlled RCTs favored manual acupuncture (3 studies) with reduced heterogeneity (mean difference, −0.88 points; 95% CI, −1.75 to −0.01; *I*^2^ = 59%) and auricular acupuncture (2 studies) with increased effect size (mean difference, −2.98 points; 95% CI, −5.37 to −0.59; *I*^2^ = 84%). By pooling the 2 open-label studies on acupressure, the effect size increased with reduced heterogeneity (mean difference, −1.75 points; 95% CI, −2.07 to −1.43; *I*^2^ = 64%) (Figure 2).

**Figure 3.**
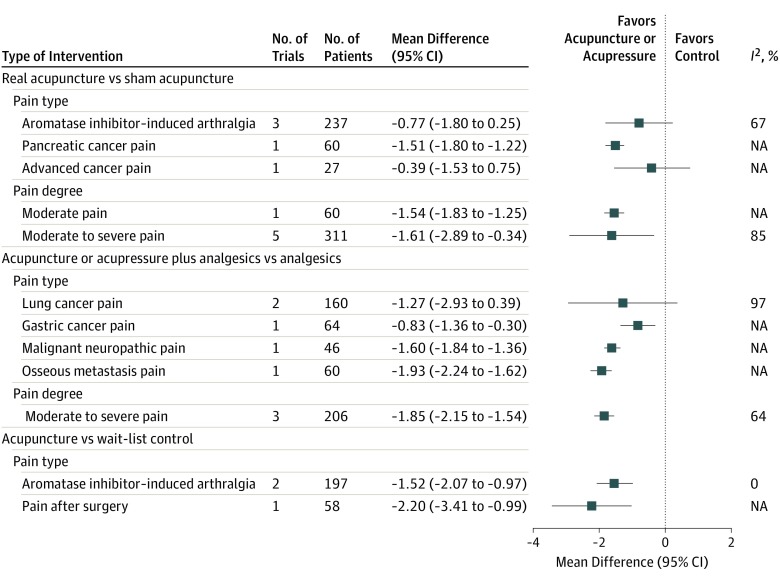
Forest Plot of the Subgroup Analyses of the Association of Acupuncture and Acupressure With Different Cancer Pain Intensity NA indicates not applicable.

In the sensitivity analyses for pain type (Figure 3), the effect sizes significantly increased for the 5 blinded trials in which pain was rated as moderate to severe with substantial heterogeneity (mean difference, −1.61 points; 95% CI, −2.89 to −0.34; *I*^2^ = 85%). In the 3 open-label studies, the effect size also increased with reduced heterogeneity (mean difference, −1.85 points; 95% CI, −2.15 to −1.54; *I*^2^ = 64%) (Figure 3).

### Safety of Acupuncture and Acupressure and Overall Evidence

The adverse events reported were minor, did not require medical evaluation or any specific intervention, and consisted predominantly of skin and subcutaneous tissue disorder or slight pain from the application of treatment to the skin. Six RCTs reported no adverse events during the study period. In all of the trials included, no dropouts were attributed to adverse effects associated with acupuncture treatment. Evidence from RCTs indicated with a moderate level of certainty that real acupuncture was associated with reduced pain intensity as compared with sham acupuncture or wait-list controls. Moderate-quality evidence also suggested that acupuncture and acupressure were associated with reduced analgesic use.

## Discussion

The systematic review included 17 RCTs involving 1111 patients with cancer, whereas the meta-analysis included 14 RCTs with 920 patients. Evidence was found of an association between real acupuncture and greater reduction in pain intensity, with a moderate level of certainty. Acupuncture may also be associated with reduced opioid use when added to analgesic therapy. Relatively few adverse events from acupuncture were reported, an observation consistent with findings in previous studies and reviews.^63,64^ The present study provides an updated synthesis of the current evidence of acupuncture and acupressure for cancer pain and identifies research gaps that remain to be addressed.

Consistent with findings of past systematic reviews and meta-analyses, acupuncture was associated with significant reductions in cancer pain in open-label studies.^23,27^ However, the present meta-analysis found acupuncture to be associated with greater pain reduction compared with sham control, which differs from findings of the previous reviews.^23,27,31^ This difference may be owing to the inclusion of recent high-quality trials.^41,48^ In addition, we applied more stringent inclusion criteria to ensure the quality of source RCTs. Considerable effort was made to conduct an extensive literature search. Seven of the 17 studies were conducted in China, 6 in the United States, and 1 study each in Australia, Brazil, France, and Korea.

The positive results from sham-controlled RCTs suggest the potential efficacy of acupuncture in reducing cancer pain, as sham acupuncture helps prevent bias in evaluating the specific outcome of acupuncture needling.^65^ Evidence from open-label studies revealed the increased risk of bias from nonblinding. However, in recent years, nonblinded pragmatic trials have been recommended for achieving clinically relevant results because of their emphasis on the practical applicability and extrapolation in real-world situations (increased external validity) over treatment efficacy.^66^ This design is particularly appropriate for researching complex and flexible interventions, such as acupuncture.^66,67,68^ It has also been suggested that pragmatic trials can provide more informative evidence for developing clinical guidelines for acupuncture.^69^ Nevertheless, a gap remains between acupuncture research and its flexible application in clinical practice.

From a clinical perspective, available evidence focuses on acupuncture as 1 component of pain management. Heterogeneity in the results suggests that the outcomes of acupuncture may be variable; thus, it may not be suitable as a stand-alone therapy for cancer pain. However, meta-analyses have reported reductions in various types of pain^70,71^ and opioid use after surgical procedures.^72^ A recent study from Italy reported the advantages of acupuncture for cancer-related symptoms in a palliative setting.^73^ However, few trials were available for certain types of pain (eg, neuropathic, osseous metastasis); thus, further research is needed to investigate the association of acupuncture with specific pain syndromes.^74,75^

How to integrate acupuncture into pain and symptom management plans for patients with cancer remains a challenge. With the move to patient-centered care^76^ and personalized medicine in cancer therapy,^77^ oncological practice and palliative care services need to provide information about treatment options and ways to access them, which can include evidence-based nonpharmacological approaches.^78^ Given that pain is a common reason that patients with cancer visit emergency departments, often followed by hospital admission,^79^ hospitals need to establish appropriate acupuncture services. With the growing evidence of the efficacy of acupuncture, most National Cancer Institute–designated comprehensive cancer centers have begun offering acupuncture.^80^ However, the cost of treatments and exclusion from insurance coverage were identified as major barriers to using acupuncture. A recent survey found that 47.9% of patients with cancer were willing to undergo acupuncture if treatments were covered by insurance.^81^ Therefore, systematic insurance coverage is needed to allow equitable access to acupuncture as part of comprehensive cancer pain management.

### Limitations

This study has several limitations. Substantial heterogeneity was observed and contributed to lowering the evidence grade from high to moderate. Sensitivity analyses were attempted through subgroup analyses, which showed reduced heterogeneity (Figures 2 and 3) for manual acupuncture and moderate to severe pain. Because cancer pain is highly complex, the type of pain, cancer treatment (eg, surgical procedure, chemotherapy, and hormone therapy as well as phase of care such as active treatment, survivorship, and palliative care), and acupuncture method are likely factors in the variability of estimates. More research in specific areas is needed to fully assess how these factors play a role in heterogeneity. Among the open-label studies, high risk of bias existed owing to lack of blinding. In most of these studies, baseline analgesic use was not specified, and in 2 studies, participants did not use analgesic therapy at baseline.^50,51^ Consequently, variations in analgesic type and dose among participants within each study and between studies also likely contributed to heterogeneity. Because of the limited number of trials included for each comparison in the meta-analysis, funnel plots were not feasible. Therefore, we could not fully evaluate publication bias.

## Conclusions

The findings of this systematic review and meta-analysis suggest that, based on moderate-level evidence, acupuncture and/or acupressure may be associated with significant reductions in pain intensity and opioid use.

## Notes

Supplement. **eTable.** Characteristics of Trials Included in the Analysis and Summary of Trial Quality Assessment
