# Supplementary material for: Clinical Evidence for Association of Acupuncture and Acupressure With Improved Cancer Pain: A Systematic Review and Meta-Analysis
Source: JAMA Oncol. 2019 Dec 19;6(2):271–8. doi: 10.1001/jamaoncol.2019.5233 (PMC6990758; doi:10.1001/jamaoncol.2019.5233)
Supplement: Supplement. — eTable. Characteristics of Trials Included in the Analysis and Summary of Trial Quality Assessment [file jamaoncol-6-271-s001.pdf]

Supplementary Online Content

He Y, Guo X, May BH, et al. Clinical evidence for association of acupuncture and acupressure with improved cancer pain: a systematic review and meta-analysis. *JAMA Oncol.* Published online December 19, 2019. doi:10.1001/jamaoncol.2019.5233

eTable. Characteristics of Trials Included in the Analysis and Summary of Trial Quality Assessment

This supplementary material has been provided by the authors to give readers additional information about their work.

©2019 He Y et al. *JAMA Oncology*

eTable. Characteristics of Trials Included in the Analysis and Summary of Trial Quality Assessment

Source*	Trial Design	Study Population	Intervention	Comparison	Pain Measure	Sample Size (A/C)[a], Dropout (A/C)[a]	Pain Degree	Age, Year (A/C) [a,b]	Gender, No. of Male (A/C)[a]	Risk of Bias[e]	Primary Outcome Result
**Hershman 2018[41] (US)**	Sham-controlled 3-arm	AIIA	MA	SA; Waitlist control	BPI	226(110/59/57), 21(10/5/6)	BPI≥3	60.8(34.1-80.6)/ 57.0(40.6-77.5)/ 60.6(27.1-76.0)	0/0/0	All low[f]	Positive[l]
**Kim 2018[48] (Korea)**	Sham-controlled 2-arm	Advanced Cancer	MA	SA	NRS	30(15/15), 3(1/2)	All degree	54.0±9.1/ 58.2±11.4	5/6	All low	Positive
**Ruela 2018[47] (Brazil)**	Sham-controlled 2-arm	Cancer pain	AA	SAA	NRS	31(16/15), 8(5/3)	NRS≥4	58.27±10.09/ 52.08±7.99	2/3	Low; unclear; low; unclear; unclear; unclear	Positive
**Wang 2017[49] (China)**	Open-label 2-arm	Non-small cell lung cancer	EA + Oxycontin	Oxycontin	NRS	60(30/30), 0	All degree	64.8±9.4/ 62.1±14.7	16/13	Unclear; unclear; high; low; unclear; unclear	Positive
**Shen 2016[50] (China)**	Open-label 2-arm	Lung cancer	EA + Three-step analgesics	Three-step analgesics	NRS	100(50/50), 0	NRS≥4	54.93±14.45/ 58.09±12.11	28/31	Low, unclear, high, low, unclear, unclear	Positive
**Guo 2015[52] (China)**	Open-label 2-arm	Gastric cancer	EA+ Three-step analgesics	Three-step analgesics	NRS	64(32/32), 0	All degree	61.5±7.6/ 60.8±8.2	20/22	Low, unclear, high, low, unclear, high	Positive
**Wang 2015[51] (China)**	Open-label 2-arm	Bone metastatic cancer	AAP + Oxycodone	Oxycodone	NRS	60(30/30), 0	NRS≥4	62.87±10.96[c]	23[c]	Low, unclear, high, low, unclear, unclear	Positive
**Mao 2014[53] (US)**	Sham-controlled 3-arm	AIIA	EA	SEA; Waitlist control	BPI	67(22/22/23), 8(3/3/2)	NRS≥4	57.5±10.1/ 60.9±6.5/ 60.6±8.2	0/0/0	All low[f]	Positive[l]
**Bao 2013[57] (US)**	Sham-controlled 2-arm	AIIA	MA	SA	VAS	51(25/26), 4(2/2)	VAS≥2	61(44-82)/ 61(45-85)	0/0	All low	Negative
**Chen 2013[56] (China)**	Sham-controlled 2-arm	Pancreatic cancer	EA	SEA	NRS	60(30/30), 1(0/1)	NRS 3-6	60.1±8.5/ 59.1±9.1	19/20	Low, unclear, low, low, low, low	Positive

©2019 He Y et al. *JAMA Oncology*

Source*	Trial design	Study Population	Intervention	Comparison	Pain Measure	Sample Size (I/C)[a], Dropout (I/C)[a]	Pain Degree	Age, Year (I/C)[a,b]	Gender, No. of Male (I/C)[a]	Risk of Bias[e]	Primary Outcome Result
**Oh 2013[55] (Australia)**	Sham-controlled 2-arm	AIIA	EA	SEA	BPI	32(16/16), 3(2/1)	BPI≥3	＜45: 12/2<br>≥45: 14/1[d]	0/0	All low	Negative
**Zhu 2013[54] (China)**	Open-label 2-arm	Malignant neuropathic	AAP + Oxycontin	Oxycontin	VAS	46(23/23), 0	NRS≥4	55.6±10.2/ 56.2±9.7	13/12	Low, unclear, high, low, unclear, unclear	Positive
**Jiang 2011[58] (China)**	Open-label 2-arm	Miscellane ous cancer	MA + AAP+ Three-step analgesics	Three-step analgesics	BPI	60(30/30), 0	All degree	20-45: 4/3<br>46-60: 17/16<br>61-75: 9/11[d]	16/17	Low, unclear, high, low, unclear, low	Positive
**Crew 2010[60] (US)**	Sham-controlled 2-arm	AIIA	MA	SA	BPI	43(23/20), 5(3/2)	BPI≥3	58(44-77)/ 57(37-77)	0/0	All low	Positive
**Pfister 2010[59] (US)**	Open-label 2-arm	Cancer pain after neck dissection	MA + Usual care	Usual care	NRS	70(34/36), 12(6/6)	NRS≥4	61(54,68)/ 57(50,63)	15/23	Low, low, high, low, low, low	Positive
**Crew 2007[61] (US)**	Open-label cross-over	AIIA	MA + Usual care	Usual care	BPI	21, 2	BPI≥3	59(46-73)[c]	0/0	Unclear, unclear, high, low, low, unclear	Positive
**Alimi 2003[62] (France)**	Sham-controlled 3-arm	Miscellane ous cancer	AA	SAA; Auricular seeds	VAS	90(29/30/31), 11(1/7/3)	VAS≥3	57(38-84)/ 56(42-72)/ 57 (37-80)	9/3/7	Low ,low, low, unclear, low, unclear[f]	Positive[l]

Abbreviations: AA, Auricular acupuncture; AIIA, Aromatase inhibitor-induced arthralgia; AAP, Auricular acupressure; BPI, Brief Pain Inventory; EA: Electroacupuncture; MA, Manual acupuncture; NRS, Numeral Rating Scale; SA, Sham manual acupuncture; SAA, Sham auricular acupuncture; SEA, Sham electro-acupuncture.; VAS, Visual Analogue Scale.

[a] A/C, data of acupuncture group/ data of control group(s);

[b] Data presented as mean (SD) or median (range);

[c] not reported separately for different groups;

[d] Data presented as the range of age: number;

[e] Risk of bias tool domains: random sequence generation; allocation concealment; blinding of participants and outcome assessors; incomplete outcome data addressed; selective outcome reporting; other potential threats respectively;

©2019 He Y et al. *JAMA Oncology*

[f] In the 3-arm studies, the risk of bias for blinding was considered low for the comparison of experimental intervention and valid sham intervention; high risk of bias for the comparison of experimental intervention and waitlist control or invalid sham control;
[l] Primary results of comparisons (experimental intervention vs. sham control(s) and experimental intervention vs. waitlist control) were both positive.

*Sources of funding for the studies included (unless not reported):
Hershman 2018: Funding was provided by the National Institutes of Health (NIH) National Center for Complementary and Integrative Health and the Office of Research on Women's Health R01AT006376; NIH/NCI/DCP (Division of Cancer Prevention) grant UG1CA189974 and legacy grant U10CA37429. The funder had no role in the design and conduct of the study; collection, management, analysis, and interpretation of the data; preparation, review or approval of the manuscript; and decision to submit the manuscript for publication.
Kim 2018: The author(s) disclosed receipt of the following financial support for the research, authorship, and/or publication of this article: This study was supported by the Bio-Synergy Research Project (NRF-2017M3A9C4065964) of the Ministry of Science, ICT and Future Planning through the National Research Foundation; and the Comprehensive and Integrative Medicine Institute (CIMI), Daegu, Republic of Korea (Grant # CIMI-15-01-07).
Wang 2017: Zhejiang Traditional Chinese Medicine Science and Technology Project (No.: 2015ZA182).
Shen 2016: Zhejiang Medical and Health Science and Technology Plan Project (2015KYA217); Zhejiang Province Medical Research Funded by the Medical Association (2013ZYC-A89); Zhejiang Province Integrated Traditional Chinese and Western Medicine Pain Medicine Innovation Project (2012-XK -22); Jiaxing Science and Technology Plan Project (2014AY21030 - 4); Jiaxing Key Discipline Respiratory Department (04 -Z-11) Oncology (04-F-14) Construction Project [Jiashi Weifa (2014) No.72].
Wang 2015: Nursing Research Project of Wangjing Hospital of China Academy of Chinese Medical Sciences (NO: WJHL2013-17).
Mao 2014: This study is supported by grants from the National Institutes of Health/National Center for Complementary and Alternative Medicine (NCCAM) R21 AT004695. Dr. Mao is a recipient of the NCCAM K23 AT004112 award. The funding agencies had no role in the design or conduct of the study.
Bao 2013: This trial was supported by ASCO Foundation Young Investigator's Award, Susan Komen Postdoctoral Fellowship Award, Breast Cancer Research Foundation, Maryland Affiliate of Susan G. Komen for the Cure Craft grant. TB is a Paul Calabresi scholar (K12 CA126849 A).
Chen 2013: This study was supported jointly by the National Natural Science Foundation of China (NSFC) (81202751 to Le Kuai), and Shanghai Science and Technology Committee (12401905600 to Hao Chen).
Oh 2013: This study was awarded the Cancer Institute NSW Innovative Grant in 2008 and supported by the Sydney South West Area Health Service, NSW Department of Health.
Crew 2010: Supported in part by a Lance Armstrong Young Investigator Award (K.D.C.) and an Advanced Clinical Research Award from the American Society of Clinical Oncology with funding from AVON Products Foundation and the Breast Cancer Research Foundation (D.L.H.).
Pfister 2010: Supported by Grant No. CA098792 from the National Institutes of Health (Bethesda, MD).
Crew 2007: Dr. Crew was the recipient of an NCI-funded postdoctoral fellowship (T32-CA09529) and a Lance Armstrong Young Investigator Award. Dr. Hershman is the recipient of a K07 Award from the NCI (CA95597). This research was funded by a grant from Women at Risk and the Avon Foundation.
Alimi 2003: This work was supported by grants from Institut Gustave Roussy (contrat de recherche clinique n°98-83), Schwa Medico, and the Lion's Club.

©2019 He Y et al. *JAMA Oncology*
